# Design and implementation of a community-based mother-to-mother peer support programme for the follow-up of low birthweight infants in rural western Kenya

**DOI:** 10.3389/fped.2023.1173238

**Published:** 2023-07-03

**Authors:** Jemma L. Wright, Florence Achieng, Linda Tindi, Manasi Patil, Mwanamvua Boga, Mary Kimani, Hellen C. Barsosio, Dan Juma, Laura Kiige, Alexander Manu, Simon Kariuki, Matthews Mathai, Helen M. Nabwera

**Affiliations:** ^1^ Department of Community Paediatrics, Countess of Chester Hospital, Chester, United Kingdom; ^2^ KEMRI-Center for Global Health Research, Kisumu, Kenya; ^3^ Department of Maternal and Child Health, Homa Bay County Referral Hospital, Homa Bay, Kenya; ^4^ Department of Education, Liverpool School of Tropical Medicine, Liverpool, United Kingdom; ^5^ Department of Clinical Research, KEMRI-Wellcome Trust Research Programme, Kilifi, Kenya; ^6^ Department of Nutrition, Action Against Hunger, Nairobi, Kenya; ^7^ Nutrition Unit, UNICEF-Kenya, Nairobi, Kenya; ^8^ Centre of Excellence for Women and Child Health, Aga Khan University, Nairobi, Kenya

**Keywords:** peer support, low birthweight infants, community based, Kenya, postnatal care

## Abstract

**Background:**

Globally, low birthweight (LBW) infants (<2,500 g) have the highest risk of mortality during the first year of life. Those who survive often have adverse health outcomes. Post-discharge outcomes of LBW infants in impoverished communities in Africa are largely unknown. This paper describes the design and implementation of a mother-to-mother peer training and mentoring programme for the follow-up of LBW infants in rural Kenya.

**Methods:**

Key informant interviews were conducted with 10 mothers of neonates (infants <28 days) from two rural communities in western Kenya. These data informed the identification of key characteristics required for mother-to-mother peer supporters (peer mothers) following up LBW infants post discharge. Forty potential peer mothers were invited to attend a 5-day training programme that focused on three main themes: supportive care using appropriate communication, identification of severe illness, and recommended care strategies for LBW infants. Sixteen peer mothers were mentored to conduct seven community follow-up visits to each mother-LBW infant pair with fifteen completing all the visits over a 6-month period. A mixed methods approach was used to evaluate the implementation of the programme. Quantitative data of peer mother socio-demographic characteristics, recruitment, and retention was collected. Two post-training focus group discussions were conducted with the peer mothers to explore their experiences of the programme. Descriptive statistics were generated from the quantitative data and the qualitative data was analysed using a thematic framework.

**Results:**

The median age of the peer mothers was 26 years (range 21–43). From March-August 2019, each peer mother conducted a median of 28 visits (range 7–77) with fourteen (88%) completing all their assigned follow-up visits. Post training, our interviews suggest that peer mothers felt empowered to promote appropriate infant feeding practices. They gave multiple examples of improved health seeking behaviours as a result of the peer mother training programme.

**Conclusion:**

Our peer mother training programme equipped peer mothers with the knowledge and skills for the post-discharge follow-up of LBW infants in this rural community in Kenya. Community-based interventions for LBW infants, delivered by appropriately trained peer mothers, have the potential to address the current gaps in post-discharge care for these infants.

## Background

Globally, 2.3 million neonates (i.e., infants <28 days old) died in 2020, and over a third of these were in sub-Saharan Africa (SSA) (1). Although significant progress has been made in reducing the mortality rate of children under 5 years old globally, the neonatal mortality rate has lagged behind and now accounts for 47% of all childhood deaths under 5 years old (1). Worldwide, 20 million newborns (up to 20% of all births) are low birthweight (LBW; <2,500 g) (2, 3). LBW is associated with adverse outcomes in the immediate newborn period and beyond (4–6). In SSA, LBW infants have a 25-fold increased risk of dying in infancy (5) and over two thirds of those who survive are stunted and/or have impaired neurodevelopment (7). Community-based interventions for LBW infants therefore have the potential to significantly reduce neonatal mortality particularly in low- and middle-income countries (LMICs), which is a key global health priority (8, 9).

The World Health Organisation (WHO) and United Nations International Children's Emergency Fund (UNICEF) both recommend home visits for newborns as a strategy to improve the survival of all newborns in the first week of life (10). However, community health workers (CHWs) are often overburdened and the communication channels between health facilities and community health teams in LMICs are inadequate (11). As a result, the post-discharge care and support of LBW infants in many LMICs remains deficient resulting in poor outcomes (6, 11, 12).

A recent study in Kenya found that accessing healthcare for LBW infants in a rural community in western Kenya was fraught with challenges at the individual, community and health system levels (13). In Uganda, a study that explored the perceptions of carers of preterm infants post-discharge found that, although carers felt that they had acquired the knowledge and skills required to care for their newborns from the facility admission, in the community they struggled to maintain that quality of newborn care in the absence of ongoing support from healthcare providers (14). In addition, they did not know how to respond when infants develop features of severe illness such as poor feeding, breathing difficulties etc. as outlined in the WHO guidance or postnatal care (15). Healthcare workers in rural health facilities in Malawi also highlighted the lack of continuity of care and follow-up in the community for the LBW infants discharged from their facilities as a key challenge (11). In Malawi, carers found that the discrimination and stigma associated with having a LBW infant was a major challenge post-discharge (11).

An international systematic review has found that community-based peer support for mothers is effective in increasing the duration of exclusive breastfeeding among term infants, encourages mothers to initiate breastfeeding early, and reduces the risk of newborn prelacteal feeding in low resource settings (16). The use of mother-to-mother peer support is therefore a potentially effective and sustainable approach to deliver a community-based package of interventions for LBW infants post-discharge.

The aim of this paper is to describe the design and implementation of a mother-to-mother peer support training and mentoring programme in rural Western Kenya. The focus of the training was to strengthen the community-based support available for LBW infants, empowering the local community with newborn care strategies to improve the health outcomes of vulnerable LBW infants.

## Methods

### Study design

**Figure 1:**
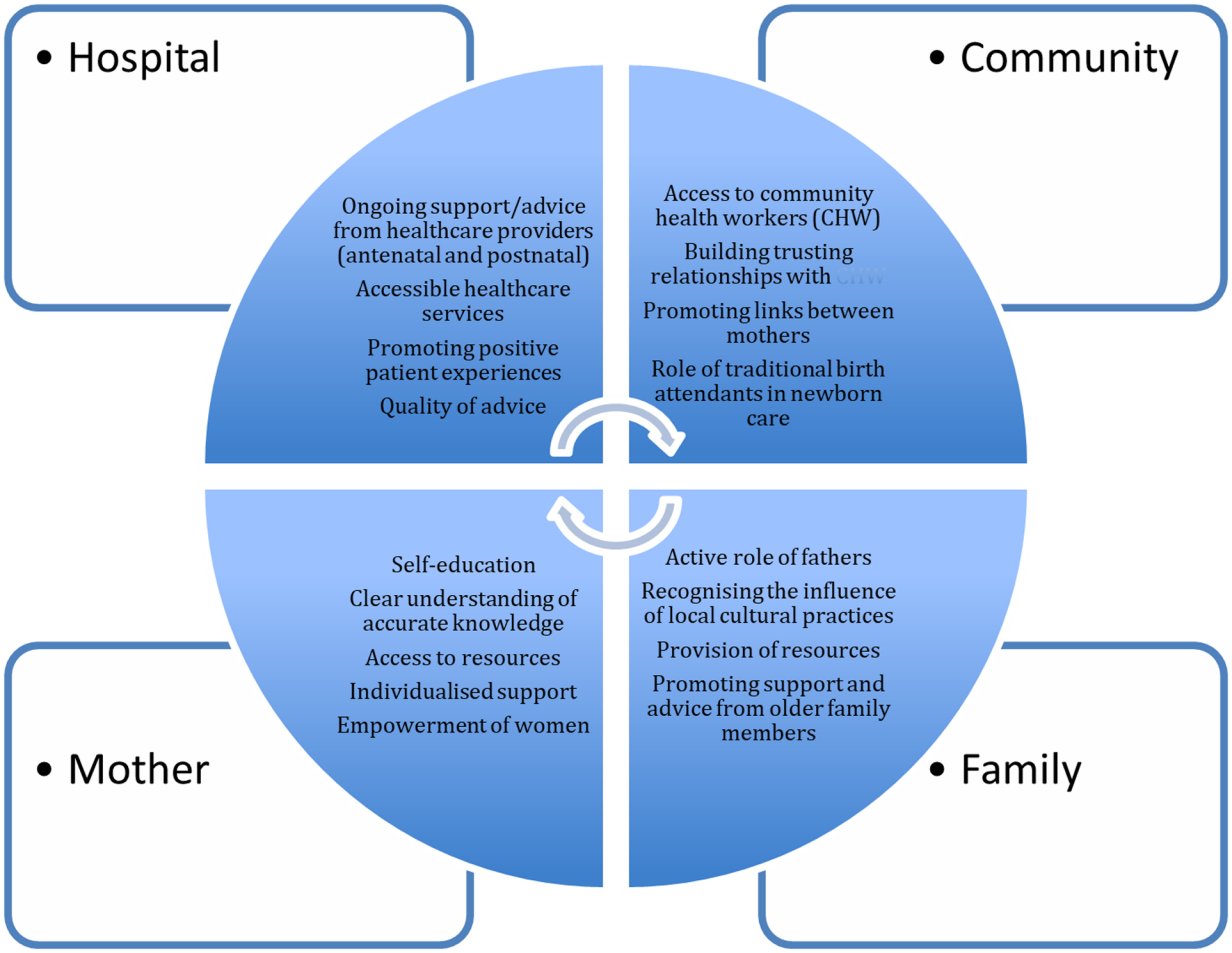
Conceptual framework to identify factors that influence the care of newborns in a rural community in western Kenya.

A mixed methods approach was employed to design and evaluate the implementation of this peer mother support training and mentoring programme for LBW infants (17). The quantitative data collection and analysis was used to evaluate the recruitment and retention of peer mothers during a 6-month feasibility study focused on the use of peer mothers in the post-discharge follow-up of LBW infants (18). Initial qualitative data from key informant interviews (KIIs) with mothers of newborn infants was used to inform the design and delivery of the training programme for peer mothers. Further qualitative data collection and analysis at the end of the implementation, using peer mother focus group discussions (FGDs), was used to explore the acceptability of the training programme. A conceptual framework was developed to guide the process of data collection and analysis (Figure 1).

### Study population

The initial study population included mothers of newborns in the rural communities of Rabuor and Ahero in Kisumu County of western Kenya. Then peer mothers from communities in the neighbouring Homa Bay County, who were recommended by the community health worker teams, were trained and recruited to follow-up LBW infants. For logistical reasons, this work was embedded within an existing maternal and child health research platform in the area meaning that the mapping of mother's experiences was conducted in Kisumu County and the peer mother support programme was implemented in Homa Bay County. All the peer mothers had cared for an LBW infant in the preceding 24 months.

### Setting

The study was conducted in the neighbouring counties of Kisumu and Homa Bay in western Kenya. Each county has a predominantly rural population of approximately 1 million (18). Nearly two thirds of the population live in extreme poverty (19). Approximately 60% of the population are affected by food insecurity (20). Less than two thirds of births are facility-based. The neonatal mortality rates in these two counties is unknown however the national mortality rate is known to be high (20 per 1,000 live births) (21). Six percent of infants are LBW with relatively high rates of undernutrition in under 5's: 5%–15% underweight, 19%–25% stunted and 4% wasted (21). Only 39% of infants under 6 months are exclusively breastfed (22).

### Sampling and sample size

#### Qualitative

At the start of the study, hospital registers at Rabuor and Ahero sub-county hospitals were used to identify women who had delivered a live newborn infant in the preceding 28 days. Purposive sampling was used to identify mothers of newborn infants with diverse characteristics for the KIIs from the communities of Rabuor and Ahero, including first time mothers, young mothers, married vs. single, and those with varying levels of education. This enabled us to explore the knowledge, perceptions, and experiences of mothers caring for newborn infants in the community. We also explored their support networks and decision-making processes around infant feeding and seeking care for their newborn infants. Purposive sampling is commonly used in qualitative research to identify a particular group of people who possess certain characteristics or who reside in circumstances pertinent to the phenomenon being studied and are therefore “*information-rich”* (23). We achieved data saturation after 10 KIIs as no new ideas were emerging from the interviews (24). This enabled us to map out maternal postnatal support structures in these communities and identify the key characteristics required in identifying the peer mothers.

At the end of the study, convenience sampling was used with the peer mothers. Peer mothers who attended the final study mentoring meeting were invited to the FGD (25), to explore their experiences of implementing the knowledge and skills that they had acquired during the training programme.

#### Quantitative

**Figure 2:**
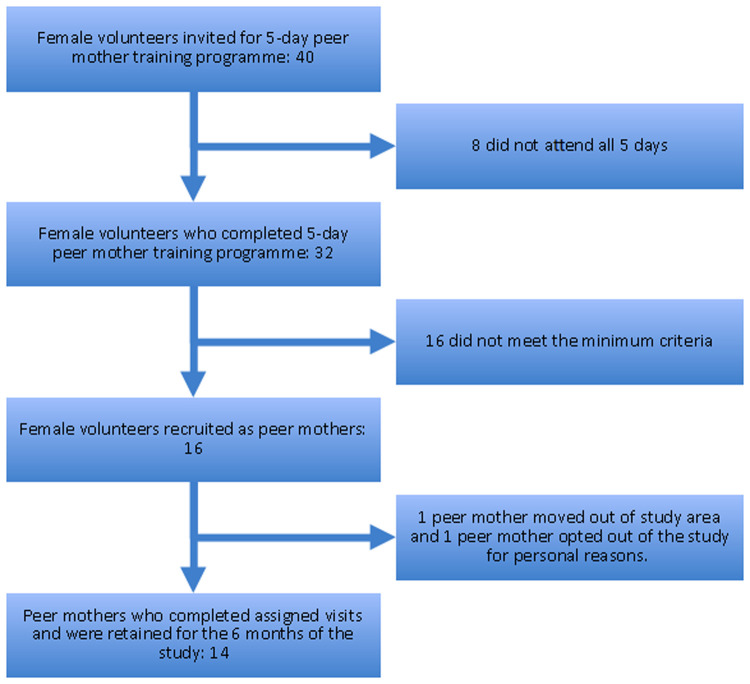
Flow chart of peer mother recruitment and retention.

We estimated that 15–20 peer mothers would be required to deliver a community-based package of interventions to 60 mother-LBW infants, aiming for 1 peer mother to support the care of 2–3 mother-LBW infant pairs in the community during the 6-month period of the feasibility study. Community health worker teams and community leaders in Homa Bay County recommended forty mothers for our 5-day peer mother training programme at Homa Bay County Referral Hospital. These mothers had all cared for an LBW infant in their communities during the preceding 24 months. These mothers also had to be able to read and write at a level equivalent of a student who had completed their primary school education. Thirty-two mothers completed the training programme but only 16 (50%) met the threshold for competency-based assessments and were recruited to deliver the community-based package of interventions (Figure 2).

### Training programme

The training programme was developed and delivered by a multidisciplinary team of collaborators including the high dependency unit nurse manager from KEMRI-Wellcome Trust programme in Kilifi, Kenya (MB), senior nutritionists from UNICEF-Kenya and Action Against Hunger, Kenya (LK, MK), and a consultant paediatrician with expertise of working in rural communities in Africa (HMN). Collectively, this group had extensive experience of working among rural communities in Africa, including previous experience of training of healthcare providers about communication skills and emotional competency, basic life support skills and emergency care in children, and infant feeding and caring strategies. The learning outcomes of the training programme were:

A) To enable the peer mothers to understand the challenges faced by LBW infants, for example difficulties feeding and increased risk of severe illness.
B) To enable the peer mothers to understand the importance of adequate nutrition and safe newborn care practices, and to enable peer mothers to conduct practical tasks (such breastfeeding positioning, kangaroo mother care, identifying a newborn needing urgent intervention by a healthcare worker, etc) on home visits in their role as peer mothers.

**Figure 3:**
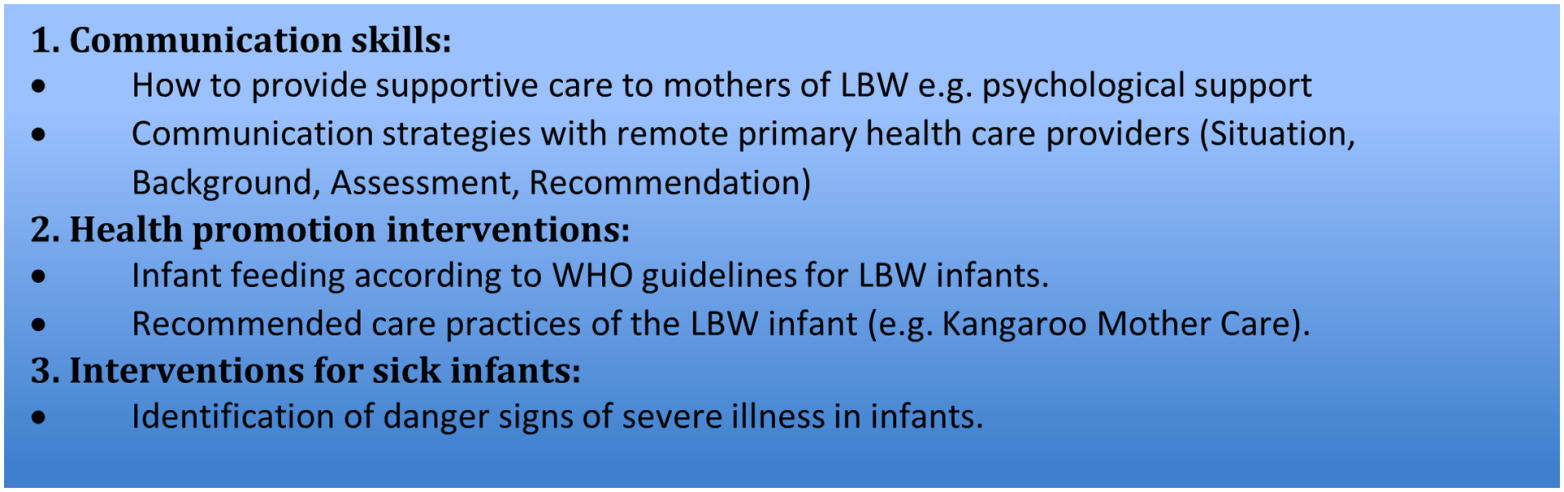
Topics covered in training programme for peer mothers.

The training programme was developed based on existing international and Kenyan national guidelines for infant feeding, essential newborn care (with particular attention to the extra requirements of an LBW infant), and postnatal care (15, 26–29). The topics covered are outlined in Figure 3. A diverse range of adult-learning techniques were used including didactic and student centred methods using PowerPoint slides, case-based discussions, role play, reflective practice, and mentorship (30). At the end of the training, all the potential peer mothers underwent a competency assessment using case-based and role play scenarios that were led by three of the trainers (MB, MK, HMN). The potential peer mothers were recruited to deliver the community-based interventions (including supporting LBW infant feeding practices, providing psychological support for mothers, and helping identify severely ill LBW infants) if they had achieved the required competencies in 9 key areas within the 3 key topics (Supplementary material). Sixteen (50%) peer mothers met these criteria and were recruited. This fell within our estimated sample size of 15–20 peer mothers. These peer mothers conducted seven community follow-up visits to each 60 mother-LBW infant pairs over a 6-month period (∼ 4 mother-LBW infant pairs per peer mother). Each peer mother was given a diary to record the main points of their visits. Peer mothers were reimbursed for any travel or childcare costs they incurred during the home visits.

### Data collection

#### Qualitative

##### Pre-training

The KIIs were conducted by a trained research assistant (FA), who originated from this region of Kenya, was a mother herself, and had over 10 years' experience in conducting qualitative interviews in this community. These interviews were conducted in the local languages (Dholuo, Kiswahili) or English depending on the language that a mother was fluent in. The KII guide was divided into the following four areas of inquiry: infant feeding; parenting skills; healthcare seeking behaviour; and support structures for mothers with newborn infants in the community. Each section had a combination of structured and open-ended questions. The interviews were audio-recorded and were complemented by field notes taken by the research assistant simultaneously.

##### Post-training

At the end of the feasibility study, two concurrent FGDs were conducted with the peer mothers. Sixteen peer mothers achieved the required competencies of the training programme, but one peer mother was absent on the day and the other one had dropped out of the study. Therefore, 14 peer mothers joined one of the two FGDs so each group had seven peer mothers present. The first FGD was convened by the research assistant, and the second one by the male field supervisor, who also had been trained in qualitative research methods. The FGD and KII guides can be found in the Supplementary Material.

##### Quantitative

Demographic details of all the peer mothers were collected as they registered for the training programme. The field supervisor (DJ) kept a record of their activities, including home visits and meeting attendance. He maintained weekly contact with them and the community health visiting teams via mobile phone communication on the days with no scheduled study visits. He also reviewed their diaries for completeness.

From March to August 2019, peer mothers had fortnightly debriefing meetings with members of the research team as well as maternal and newborn senior nurses at Homa Bay County Referral Hospital. During these meetings, they gave updates on the mother-infant pairs who had been assigned to them. These meetings also focused on the challenges and successes of facilitating shared learning, problem solving, team building, and enhancing peer support.

### Data management and analysis

#### Qualitative

The interviews in Dholuo or Kiswahili were translated and transcribed concurrently by the research assistant. The interviews in English were transcribed verbatim by the same transcriber. The transcripts were reviewed for translation accuracy by the research assistant and discussed with the principal investigator (HMN) during debriefing sessions. Any problematic translations in the transcripts were compared to the original audio recordings and revised accordingly. NVivo 12 software (QSR International Pty Ltd 2012) was used to manage and support the analysis of the data. A thematic framework approach was used to analyse the data (31). This involved initial familiarization with the data and generation of codes by five study members (HMN, FA, KM, JW and MP) to enhance the credibility of the findings. The data was coded separately by two study members (JW and MP) and any inconsistencies were discussed with the principal investigator. Key themes were identified and agreed upon by three of the co-authors (JW, MP and HMN) (32). Inductive coding was used where codes emerged from a thorough review of the data (33).

#### Quantitative

Anonymized data were manually recorded onto the data collection forms and then entered onto an Excel spreadsheet that was stored on the secure LSTM server. Data was imported from Excel to Stata 15 (StataCorp., Texas, USA) for analysis. Descriptive statistics, including mean (standard deviation), median (interquartile range), and number (percentage), were used to describe the recruitment, training, characteristics, activity, and retention of peer mothers.

### Ethics approval

The project was approved by the Research and Ethics Committee at the Liverpool School of Tropical Medicine (protocol number: 18–076) and the Kenya Medical Research Institute Scientific and Ethics Review Unit (KEMRI-SERU)) (protocol number: 3760). Written informed consent was obtained from all participants including the peer mothers prior to recruitment. Participants were given the option of consenting on the same day or after 24 h following consultation with other decisions-makers in their household. It was emphasised that consent was voluntary, and all participants were informed that they could withdraw from the study at any point without providing a reason. Anonymity was maintained by the study team and any identifiable details were removed from the recordings during the transcription process.

## Results

### Qualitative (pre-training)

The pre-training qualitative data informed the focus areas for the peer mother training, including communication skills, breastfeeding, thermal care, hygiene, and early identification of danger signs (i.e., features of severe illness in the infants such as poor feeding and breathing difficulties). The baseline mapping interviews were conducted with 10 mothers of newborn infants in the rural communities of Rabuor and Ahero in western Kenya. Their mean age was 27 years (range 20–34 years). Educational level varied with one mother having college education whilst the rest had secondary school education. Seven of the mothers were married, two were widowed, and one was single. The mean number of children was 2 (range 1–4 children).

There were 4 key themes that emerged from the interviews about their experiences of community-based support for newborn care.

#### 1. Variable knowledge and understanding of newborn feeding and care practices

There was a variable knowledge amongst the mothers when they were interviewed about infant feeding and hygiene practices. The majority of mothers reported that exclusive breastfeeding and appropriate hygiene practices were best for their infants as this was the advice that they had received at the hospital. However, they were often not clear about the details of this received advice (Table 1: Quote 1). This suggests that there was a lack of clear explanation provided to these mothers by healthcare workers about how to breastfeeding and provide other care for their infants, as well as where and when to seek help.

#### 2. Sources of advice on newborn feeding and care practices varied depending on access to healthcare providers

Most of the advice about infant feeding and care practices was given to the mothers by healthcare providers during their pregnancy or at the time of birth. The mothers felt that this was a trusted source of advice (Table 1: Quote 2). Many mothers were reserved when commenting about their experience with the healthcare providers. However, some mothers reported that relevant information was not provided when they attended health facilities and that they did not have access to ongoing support after they were discharged from hospital (Table 1: Quote 3 and 4). Community health workers (CHW) were a valuable source of support and advice for some of the mothers of newborn infants (Table 1: Quote 5). However, other mothers were concerned about the limited training that CHWs had received so they felt that their advice was less trustworthy (Table 1: Quote 6). Interestingly, some mothers used sources of advice from the community. These include other traditional birth attendants, other mothers, and older women in the family (Table 1: Quote 7 and 8).

#### 3. Disconnect between knowledge of newborn care recommendations and practice in the community

Most of the mothers followed the advice to exclusively breastfeed their infant for six months, however some highlighted that this advice was not in line with the feeding practices followed by other mothers in their community. Mixed feeding of newborn infants was common with some mothers starting their newborn babies on solids from 2 weeks onwards due to the perception of having insufficient breast milk (Table 1: Quote 9 and 10). Mothers also described the challenges that their poor living conditions and inadequate resources posed to maintaining good hygiene when caring for their newborns (Table 1: Quote 11). Despite this, poor hygiene was frowned upon by most of the mothers and attributed to “laziness” amongst mothers of newborn infants (Table 1: Quote 12).

#### 4. Mothers were the primary carers of newborn infants but require support from the wider family

In this community mothers were the primary carers of newborn infants. However, mothers also reported that other family members did offer some hands-on support (Table 1: Quote 13).There was recognition by many of the mothers that the father provided financial and emotional support to the mother and baby. Beyond being a provider, mothers reported that they appreciated the hands-on support with infant care that they received from some fathers in the context of the multiple demands on their time in the home. (Table 1: Quote 14 and 15).

**Table 1:** Pre-Training themes.

Theme	Quote	
Variable knowledge and understanding of newborn feeding and care practices	Q1	“They only told me to breastfeed, but they did not tell me for how long.” [Mother A]
Sources of advice on newborn feeding and care practices varied depending on access to healthcare providers	Q2	“You know the hospital is good. Anything that the doctor tells you is very good. So, I just try and follow the doctor's advice.” [Mother B]
	Q3	”They (healthcare providers) did not tell me anything (advice on how to feed the newborn in hospital or after discharge).” [Mother H]
	Q4	“They (healthcare providers) are always very harsh; you cannot even ask them (advice on how to care for their newborn).” [Mother C]
	Q5	“When you go to the CHW for advice, she can tell you how to take care of the baby in terms of the hygiene and how you can stay with the child.” [Mother F]
	Q6	“Maybe CHW's…But I do not support that fully because not all of them have been trained.” [Mother G]
	Q7	“When you do not go to the hospital you will now go to the TBA (Traditional Birth Attendant).” [Mother F]
	Q8	“I think it is only the elderly who have already had children who know how to take care of a baby… only those who know how to take care of a baby…There are some who are elderly and have had babies but they do not know how to take care of babies. That is why I say that the elderly who know how to take care of babies.” [Mother J]
Disconnect between knowledge of newborn care recommendations and practice in the community	Q9	“Some start feeding their babies porridge at two weeks. They say that they do not have enough milk, so they start (to) prepare (for) it (baby) porridge.” [Mother I]
	Q10	“Sometimes when you only give breast milk, she cries.” [Mother E]
	Q11	“Sometimes you do not have soap, you cannot get it from anywhere and there is no one who can give it to you.” [Mother B]
	Q12	“It is only when someone is lazy that they can let the house to be untidy.” [Mother J]
Mothers were the primary carers of newborn infants but require support from the wider family	Q13	“If one is married, the husband can help you and if you are a young girl, your mother can tell you what to do if she is around.” [Mother F]
	Q14	“When you both agree, the father can take some time to take care of the baby…He comes back (from work) very tired and cannot look after the baby…When you are also tired because the baby is crying too much, you can ask him to carry her… he can carry her for, I know only for thirty minutes then he will tell me to take her again. He can do it only for a short time.” [Mother B]
	Q15	“He can show the baby some love, make her happy.” [Mother J]

### Quantitative (training, recruitment, and retention)

The quantitative data provided information about the key characteristics of the trained peer mothers and their follow-up of the mother-LBW infant pairs post-discharge.

The median age of the peer mother was 26 years old (range, IQR 21–43). Eighty percent of the peer mothers were married. All peer mothers had a basic level of education with 60% having primary level education and 40% having post-secondary level education. The majority (53%) of the peer mothers stated that their primary source of income was farming with 27% employed in a small-scale business and 20% employed as a casual labourer or stating that they were a housewife (Table 2).

**Table 2:** Peer mother sociodemographic characteristics.

Characteristics	*N* = 15*
Age, years (median, interquartile range)	26 (21, 43)
Married, *n* (%)	12 (80)
Education, *n* (%)	
Primary level education	9 (60)
Secondary level education	0
Post-secondary level education	6 (40)
Source of income, *n* (%)	
Farming	8 (53)
Small scale business	4 (27)
Housewife/Casual labourer	3 (20)

* 15 peer mothers were included at the end of the study.

The peer mothers were allocated a median of 5 (range 0, 11) LBW infants during the 6 months period, resulting in each peer mother conducting a median of 28 (range 7, 77) home visits. The activities of the peer mothers were monitored using their diaries, which showed a high level of completeness (93%) during the first three months. The completeness of the diaries dropped at 3 months to 79%, which suggests their activity lessened as time progressed (Table 3).

**Table 3:** Peer mother activities over the 6 months period.

Activities	Median (range)
Number of infants allocated to each peer mother	5 (0, 11)^+^
Number of follow-up visits conducted by each peer mother over 6 months	28 (7, 77)
All sections of diary completed on each home visit (One diary from each of the 14 peer mothers who completed the study reviewed)**	*n* (%)
24 h	13 (93)
Day 3	13 (93)
Day 7	13 (93)
Day 14	14 (100)
Day 28	14 (100)
2 months	13 (93)
3 months	11 (79)

** Blank copy in Supplementary Materials.

^+^ One peer mother left the catchment area soon after training/recruitment and returned in the last month of the study so, she was paired up with another more experienced peer mother for the follow-up visits. She did not go to any home visits alone.

At the end of the study, fourteen peer mothers were retained and completed a minimum of seven post-discharge home visits per mother-infant pair. More details of these visits are in a related manuscript that is due to be submitted (18) (Figure 2). Over the 6 months duration of the study, 88% of peer mothers were retained and they completed multiple follow-up visits with no adverse outcomes.

### Qualitative (post-training and after delivering package of interventions)

The post-intervention FGDs aimed to explore the perspectives of the peer mothers at the end of the training/mentoring programme. The 3 key themes from the post intervention discussions are described below.

#### 1. Peer mothers promoted appropriate feeding practices among LBW infants due to the training that they had received

The peer mothers reported that the training improved their understanding of appropriate infant feeding practices (Table 4: Quote 16). This knowledge enabled peer mothers to encourage mothers of LBW to optimise their breastfeeding, resulting in improved growth of their babies (Table 4: Quote 17 and 18). Poor breastfeeding and complementary feeding practices were still widespread in the wider community. Complementary foods were often introduced at the wrong time and were often nutritionally inadequate and unsafe. However, the peer mothers were able to provide practical support for local mothers regarding breastfeeding, such as providing advice about attachment, positioning, and maternal diet (Table 4: Quote 19 and 20). The peer mothers also reported that they helped reduce the local cultural stigma related to expressing breastmilk in the community, which was beneficial for feeding LBW infants (Table 4: Quote 21 and 22).

#### 2. Increased knowledge of LBW infant care practices coupled with lived experiences enabled peer mothers to enhance uptake of recommended practices among mothers

Peer mothers reported improved knowledge and confidence in supporting mothers of LBW infants in the community, including specific care strategies such as Kangaroo Mother Care and cleanliness (Table 4: Quote 23 and 24). Regular contact from the peer mothers during repeated individual visits enhanced the uptake of recommended infant care practices among mothers of LBW infants (Table 4: Quote 25 and 26). The lived experiences of the peer mothers enhanced the authenticity of their support for mothers of LBW infants and they were therefore well accepted by their communities (Table 4: Quote 27). Some local mothers did not accept the teaching of the peer mothers if it was not supported by their wider family and local cultural traditions (Table 4: Quote 28). However, the fact that the peer mothers were integrated into the wider healthcare team helped them to be accepted better (Table 4: Quote 29). Our results provided numerous examples of positive changes in infant care practices among mothers of LBW infants in the community (Table 4: Quote 30, 31 and 32).

#### 3. Peer mother support promoted resilience among mothers with LBW infants against community misconceptions

The stigma of having an LBW infant was highlighted by many of the peer mothers (Table 4: Quote 33). However, the peer mothers often used their own personal experience of raising an LBW infant to encourage mothers of LBW and their family members to prioritise the care of these vulnerable infants to improve their chances of survival (Table 4: Quote 34).

**Table 4:** Post-Training themes.

Theme	Quote	
Peer mothers promoted appropriate feeding practices among LBW infants due to the training that they had received	Q16	“Children with low birth weight should only be given breastmilk. The attachment and positioning of the baby should be checked for the baby to breastfeed well …. Some women say breast milk is not enough, so the child should be breastfed after every two hours. Frequent breastfeeding will help in stimulating the milk secretory glands to secrete more milk.” [FGD1 Peer Mother 4]
	Q17	“There was a mother of twins who complained that her babies were not breastfeeding to their fill because her babies used to cry a lot. I told her that the reason could be that she wasn’t breastfeeding them as often as she could. She took my advice and started breastfeeding them after every 2 h and they were now being satisfied. The woman I’ve visited only breastfeed their babies.” [FGD1 Peer Mother 4]
	Q18	“Mine (the mother I supported) was happy that her babies were gaining weight because she thought that her babies would not grow as big as they were. I told her that breastfeeding them was the cause of this growth and that motivated her to continue following the teaching.” [FGD1 Peer Mother 2]
	Q19	“When the mother is not processing enough milk, she is told what to eat, the kinds of food she should eat like vegetables. She is also advised to be stress free because that will make her feed her baby well.” [FGD2 Peer Mother 6]
	Q20	“I found a grandmother whose daughter had died. She was feeding the baby milk with a spoon. I advised her to give milk to the baby via a cup and when I can back, I found her doing just that and the baby had gotten used to using a cup.” [FGD1 Peer Mother 3]
	Q21	“They never heard of expressing milk. According to their culture, when you pressed milk while the baby was alive then you wanted to kill the baby…As we walked around and taught them, they at least got the knowledge and understanding of the importance of pressing milk. When they followed this, their children grew healthy and did not die.” [FGD2 Peer Mother 7]
	Q22	“These teachings eradicated the stigma from the cultural beliefs that expressing milk is associated with the death of children. The teachings reduced the stigma and the mother is encouraged to feed the baby using this method well for its good growth.” [FGD2 Peer Mother 6]
Increased knowledge of LBW infant care practices coupled with lived experiences enabled peer mothers to enhance uptake of recommended practices among mothers	Q23	“Children born with low birth weight require more intensive care compared to these other normal babies.” [FGD2 Peer Mother 5]
	Q24	“Cleanliness should be maintained around a child with low birth weight to prevent disease causing germs because its immune system is still weak.” [FGD2 Peer Mother 2]
	Q25	“She will take time to adapt to your new teachings. She will pretend that she is doing it and then next time you will find her not doing it so, you have to do it practically for her to see and you should also do a follow up to ensure she continues with the teachings. It always takes them time to adapt.” [FGD2 Peer Mother 6]
	Q26	“In hospital they are taught as a group and not individually. She might take the teaching shallowly…When the teachings are given to individuals, they are well and easily understood compared to when they are given publicly or to a group.” [FGD2 Peer Mother 5]
	Q27	“When I told them that my baby was also born with low birth weight of 1.8 kilos and the other 2.1 kilos, they accepted me with open arms since they saw how they had grown.” [FGD2 Peer Mother 4]
	Q28	“Some did not follow what was taught in the hospital because they heed to their grandmother's teachings. So, they were against the hospital teachings unless their grandmothers agreed to them.” [FGD1 Peer Mother 7]
	Q29	“She was informed that someone would pay her visits so when I visited her, she agreed to follow my teachings because she was told I would be sent to her to help her.” [FGD2 Peer Mother 5]
	Q30	“When I taught her how to maintain cleanliness and hygiene, she started practicing it and her cleanliness improved.” [FGD1 Peer Mother 7]
	Q31	“It was good because it helped them; how we taught them how to feed the child, “kangaroo” and taking good care of the baby can boost the child's growth. When they did this, they were happy and proud of how their children had grown.” [FGD2 Peer Mother 7]
	Q32	“She noticed it helped her because her child has not fallen sick ever since she was discharged from hospital. Yeah, it helped her because she feels good to live in a clean environment.” [FGD1 Peer Mother 5]
Peer mother support promoted resilience among mothers with LBW infants against community misconceptions	Q33	“People think that children born with LBW are sick and will not grow. Most people think they are born with a sickness or even are cursed that is why they are born when they are tiny.” [FGD1 Peer Mother 2]
	Q34	“I told her that she was not the only one who gave birth to a low-birth-weight baby. I shared my experience with my baby and told her how mine grew big and strong and encouraged her that hers too will grow big and healthy.” [FGD1 Peer Mother 5]

## Discussion

Our findings showed that the 5-day training programme for peer mothers, complemented with mentoring and debriefing sessions, equipped them with the knowledge and skills required to support the post-discharge care of LBW infants in western Kenya. The peer mothers were well accepted by their community and reported specific examples of improvements in the infant care practice and health seeking behaviour of other local mothers. Our results therefore suggest that the training of peer mothers was beneficial for the wider community. A recently published systematic review that evaluated interventions to improve the survival of preterm infants in LMICs found that low-cost interventions, including feeding support and thermal regulation, were key factors in the improved survival of these infants (34). Furthermore, community mobilisation, using strategies such as community education packages, was an effective way to deliver these interventions (34). Although community-based peer support has previously been shown to promote optimal breastfeeding practices among term infants in LMICs (reducing the risk of non-exclusive breastfeeding by 29%–37%) (16, 35, 36), this has not yet been demonstrated amongst mothers of LBW infants to our knowledge.

Our baseline data from mothers of newborn infants showed that, although healthcare providers were a trusted source of advice for infant feeding and care practices, they were not easily accessible to mothers when they were back in the community post-discharge. Mothers therefore sought advice from multiple sources due to uncertainty about the advice that they had received. Interestingly, even the advice from community health workers was not always trusted. Our study also showed that home visits to LBW infants by skilled peer mothers with links with trusted healthcare providers led to positive interactions and better uptake of recommended infant feeding and care practices. A recent meta-analysis of postnatal care in sub-Saharan Africa showed that women living in rural areas with low literacy levels were less likely to utilise postnatal care services (37). This is likely to be worse among mothers of LBW infants, due to the stigma of having an LBW infant, which can result in a lack of prioritisation of the health needs of these infants within households (5, 13). The regular contact with peer mothers enhanced the uptake of the recommended LBW infant feeding and care practices by intensifying the interventions and building resilience amongst the mothers. This regular support is particularly important in the contexts where women have lower levels of literacy (38).

The attrition of peer mothers was minimal showing that the training and mentoring programme, coupled with their lived experiences, enhanced their motivation. This helped to mitigate the psychological distress of providing community-based support for infants at high risk of adverse outcomes. A systematic review exploring the motivating factors among community health workers for maternal, newborn, and child health service delivery in LMICs found that individual motivating factors at the outset improved retention although the health system challenges in these settings demotivated them (39). Indeed, attrition rates for community health workers in Africa are >20% after 5 years of service due to inadequate renumeration, lack of career progression, and heavy workload (40). The short 6-months duration of the project may have contributed to our low attrition rate. There is the potential to scale up and enhance the sustainability of this peer mother training programme by having 6–12 monthly cycles of training in future. This would enable peer mothers to mentor other peer mothers, thereby disseminate knowledge and skills about the care priorities for LBW infants in the community, without burdening a small number of mothers for prolonged periods. The skills that peer mothers obtained during this programme may provide opportunities for them to pursue further training and job opportunities in the health sector, which was the case for some of our peer mothers. Literature from high-income settings suggests that volunteering could improve future career prospects for individuals (41); however, there is no relevant literature available in African settings. The potential benefits of volunteering requires further exploration in LMIC settings where the discourse around female volunteers often focuses on the negative socio-economic impact on women (42).

### Strengths and limitations

A key strength of this training programme was the use of adult learning approaches (30). The programme involved a combination of didactic teaching and hands on practical sessions. Case-based teaching, included case scenarios with feedback from peers, helped the peer mothers apply their knowledge and reflect on their performance during the debriefing sessions. These approaches have been used successfully to enhance communication skills among newborn care units and healthcare providers in Kenya (43). To our knowledge, this was the first time that this approach was used to train lay people in Kenya. Previous training programmes for community-based volunteers have focused on testing of knowledge alone with limited exploration of how the volunteers applied that knowledge and the challenges they faced (44).

The main limitation of this training programme was that, although the skills and knowledge evaluation strategies incorporated have been used in training healthcare providers in Kenya and The Gambia, they have not been validated for use among peer mothers. Further refinement of these strategies is work in progress. To enhance the skills and knowledge of the peer mothers, they were provided with ongoing mentoring and regular debriefing sessions. This meant the peer mothers were able to ask questions about any challenges they faced with their instructors in order to identify appropriate solutions. This also provided the opportunity for the peer mother's competencies to be reviewed by their instructors at least every 2 weeks depending on the needs of the peer mother. We plan to make the training resources widely accessible so that other researchers/educators in other LMICs can access them and evaluate their use in different contexts. Finally, the travel costs and childcare costs that the peer mothers incurred during these home visits were reimbursed, which may have contributed to the low attrition rate that may not be replicated in the “real life setting” where these costs are not reimbursed consistently or in a timely manner.

## Conclusion

Our peer mother training and mentoring programme appeared effective in enabling peer mothers to attain the competencies required to enhance the post-discharge follow-up of LBW infants in this rural community of western Kenya. In contexts where community health workers are overwhelmed with multiple priorities for maternal and child health, and therefore are unable to provide adequate post-discharge care to these vulnerable infants and their mothers, this peer mother programme has the potential to address this gap. Further refinement and evaluation of this programme in different contexts in sub-Saharan Africa is required to facilitate scale-up.

## Data Availability

The raw data supporting the conclusions of this article will be made available by the authors, without undue reservation.
