# Supplementary material for: Design and implementation of a community-based mother-to-mother peer support programme for the follow-up of low birthweight infants in rural western Kenya
Source: Front Pediatr. 2023 Jul 3;11:1173238. doi: 10.3389/fped.2023.1173238 (PMC10352086; doi:10.3389/fped.2023.1173238)
Supplement: Supplementary file 1 [file Datasheet1.pdf]

Supplementary Material:

S1: Mapping Key Informant Interview Guide for Mothers

[Before turning on the recorder]

- Introduce yourself
- Go through the information leaflet and consent form (consent form completed prior quantitative questionnaires)
- Go over areas to cover
- Explain that you will note down anything that you want to come back to
- Reiterate that there is no right or wrong answer
- That all the information that she gives will be kept confidential and will only be shared with those involved in the study
- That the data collected will be anonymised so they will not be identified
- That any information that she provides that indicates she or someone else is at risk of harm will have to be shared with the relevant health care providers (Ahero/Rabuor) for hers and the child's or other adult's safety.

Introduction: I have come to discuss with you about your experiences of looking after a newborn baby in this community and to find out what support is available for mothers of newborn babies in this community.

Infant Feeding Practices:

- What is your understanding of how a newborn baby should be fed?
  - How are you feeding your baby?
  - How did you decide?
- How do other mothers in this village feed their newborn babies?
- Where do mothers get advice on how to feed their newborn babies?
  - Where did you get your advice from?
- What factors influenced the choices that you made with regard to feeding your baby?
  - Were there any factors that limited your choices?
- Tell me about your experience with health care workers during pregnancy, delivery and now?
  - Was the infant feeding information from health care workers available to you?
  - Was it helpful? Can you give me an example?
  - What factors prevented you from adhering to this information?

Hygiene:

- In your view, how does hygiene in the household affect the health growth of your baby?
- What are the challenges of maintaining good hygiene in this environment?
  - What factors limit a mother's/carer's ability to maintain hygiene in her household? How?
  - Do these also affect her ability to keep the newborn baby clean?
- Where do mothers get advice from regarding the hygiene of their babies and households?
  - Is it useful?

Parenting Skills:

- Who is primarily responsible for looking after newborn babies in this community?
- What is the role of fathers in the care of newborn babies in this community?
- Who would mothers go to for advice on caring for their newborn babies in this community?

Is there anything you would like to ask me?

Thank you for your time

[Turn off the recorder]

Debrief:

- Inform the participant that findings will be fed back to the community and made public after the study has been completed

Once the participant has left or you have left the participant's compound, please make **field notes**:

- Any notable themes
- Social characteristics of setting
- Participant characteristics
- Your perception of the person/thoughts/emotions
- Notable events during interview
- Note any suggested changes to the topic guide

S2: Post-intervention Focus Group Discussion with Peer Mothers

[Before turning on the recorder]

- Introduce yourself
- Go through the information leaflet and consent form (consent form completed prior quantitative questionnaires)
- Go over areas to cover
- Explain that you will note down anything that you want to come back to
- Reiterate that there is no right or wrong answer
- That all the information that she gives will be kept confidential and will only be shared with those involved in the study
- That the data collected will be anonymised so they will not be identified
- That any information that she provides that indicates she or someone else is at risk of harm will have to be shared with the relevant health care providers (Ahero/Rabuor) for hers and the child's or other adult's safety.

Introduction: I have come to discuss with you all in more details the issues around your experiences of providing support to mothers with smaller than average weight (i.e. low birth weight weighing less than 2,500g) in this community.

Infant Feeding Practices:

- How do mothers in this community feed their newborn babies?
  - Where do they get advice on how to feed their infants?
- What is your understanding of how a low birth weight baby should be fed?
  - From your home visits with mothers, how did they feed their babies?
  - How did they make those choices? Why?
  - In you view, what helped them the most in following the recommendations?
  - What did mothers find most challenging when feeding their babies?
- Had the mothers heard of expressing breast milk to feed your baby?
- Please share your experiences of supporting mothers to do this?
  - How did you/they find it?
  - Did they find it difficult to do on a regular basis? Why?
  - How did you support them to get around that problem?
- Had they heard of using a cup to feed their babies if they were not able to breastfeed?
  - Please share your experiences.
  - How they find it?
  - What were the challenges of doing this?

        - How did you support them?
- What motivated them to continue breastfeeding or expressing breastmilk and giving it by cup to their baby?
    - Was the infant feeding information from health care workers available?
    - Did they find it helpful? Can you give me an example?
- What factors prevented them from adhering to this information? How?

Hygiene:

- In your view, how does hygiene in the household affect the health and growth of babies?
    - What aspects are particularly important? Why?
- What are the challenges of maintaining good hygiene in this environment?
- How does hand washing affect a baby's health and growth?
- Does the availability of clean water and good toilet facilities in the household have an effect on the health and growth of your baby? How?
- What factors limit a mother's/carer's ability of maintaining hygiene in her household?
    - Was this your experience with mothers in the study?
    - Did they find your support helpful with maintaining the hygiene of their baby?
        - How?
        - How could this have been done better?

Parenting Skills:

- Who is primarily responsible for looking after newborn babies in this community?
    - What was your experience during the home visits?
- Did any of them practise "Kangaroo Mother Care" (skin to skin contact with baby over mother's or another responsible adult's chest)?
    - Were there any challenges?
    - Did they find your support helpful? How?
    - What could have been better? How?
- What factors limited/limit their ability to care for your baby?
    - How?
    - What would have helped?
- What is the role of fathers in the care of newborn babies in this community?

        - Were your babies' fathers involved in their care? How?
        - What prevented them from getting involved or doing more?
- In your view, do you think having fathers more involved in the day to day care of newborn babies would improve their health and growth?
- How does the structure a family affect a newborn's health the growth e.g. polygamy (co-wives), living with grandparents etc

Accessing Care:

- How did you deal with health concerns among the babies who you visited at home?
    - Did you consult a health care worker?
        - Was this useful? How?
        - What challenges did the carers experience in accessing care for their newborn babied?
    - Did they find your support helpful? How?
    - What could have been better? How?

Miscellaneous:

- Have any of you experienced the death of a newborn?
    - How did you/they manage?
    - What do you think would have made a difference? How?
    - In your view, did the mothers/carers find your support helpful?
        - How?
- What could you have been better?
- What factors influenced the choices that you made regarding feeding your newborn baby?
- Were there any factors that limited your choices? How?
- Tell me about your experience with health care workers in Ahero and Rabuor?

Is there anything you would like to ask me?

Thank you for your time

[Turn off the recorder]

S3: Check for assessing competencies post-training

CHECKLIST FOR ASSESSING COMPETENCIES

Competencies	What they did well	Areas to improve
**Communication skills**		
1. Creating rapport		
2. Use of key communication skills: Open ended questions, non-verbals, reflecting back, listening, empathy, non-judgment etc		
3. Management of own emotions		
**Health promotion**		
1. Giving right information: KMC, breastfeeding, hygiene		
2. Positioning/ Attachment for breastfeeding		
3. Identify cause of poor feeding and finding solution		
4. Demonstrates and practices handwashing correctly		
**Danger signs**		
1. Identification of danger signs in a low birthweight baby		
2. Knowing what to do if danger signs present		

S4: Peer Mother Diaries

Development and feasibility of a community-based package of interventions using peer mothers to improve survival in rural Kenya infants after hospital discharge

**Visit timing**	**Date of visit** dd/mm/ yyyy	**Please select the most appropriate response after each visit**				Any other comments
		**A** Baby well (no danger signs)	**B** Baby not well (atleast one danger sign)	**C** Called health care worker with concerns	**D** Requested mother to go to health facility for medical review of baby	
24 hours						
Day 3						
Day 7						
Day 14						
Day 28						
Age 2 months						
Age 3 months						

Diaries for peer mothers

Mother's study ID: ☐☐ - ☐☐☐ Infant's study ID: ☐☐ - ☐☐☐ - ☐☐ Peer mother's name: ______________________________
